# Reference data among general population and known-groups validity among hypertensive population of the EQ-5D-5L in Vietnam

**DOI:** 10.1007/s11136-021-02959-2

**Published:** 2021-08-09

**Authors:** Vu Quynh Mai, Kim Bao Giang, Hoang Van Minh, Lars Lindholm, Sun Sun, Klas Göran Sahlen

**Affiliations:** 1 Center for Population Health Sciences, Hanoi University of Public Health, Hanoi, Vietnam; 2 Department of Epidemiology and Global Health, Umeå University, Umea, Sweden; 3 Hanoi Medical University, Hanoi, Sweden; 4 Research Group Health Outcomes and Economic Evaluation, Department of Learning, Informatics, Management and Ethics, Karolinska Instiutet, Solna, Sweden; 5 National Institute of Health Sciences, Bach Mai Hospital (NIHS), Hanoi, Vietnam

**Keywords:** EQ-5D-5L population norms, EQ-5D-5L reference data, EQ-5D-5L validity, EQ-5D-5L known-groups validity

## Abstract

**Purpose:**

This study aims to provide EQ-5D-5L population norms among the general population in Vietnam and to test EQ-5D-5L’ construction validity among people living with hypertension there.

**Methods:**

Descriptive statistics of the five dimensions and five levels, EQ-VAS and EQ-5D-5L indexes were categorised into gender and age groups for the EQ-5D-5L population norms. Known-groups testing was set for lower EQ-5D-5L outcomes among people who were aware of their hypertensive status, females, people with more comorbidities, less education, older ages, and higher body mass indexes. Level of confident interval was 95%.

**Results:**

The mean EQ-VAS and EQ-5D-5L indexes were 81.10 (SD: 13.35) and 0.94 (SD: 0.09) among the general population. The EQ-5D-5L outcomes were better among younger people, males, people with more education, employees, and single people. Respondents reported fewer problems with self-care and usual activities and tend to have problems at higher levels across older ages. The known-group testing showed statistically significant results. The mean EQ-VAS and EQ-5D-5L indexes of people in the diagnosed hypertensive group (71.48 and 0.94, respectively) were statistically significantly smaller than they were in the non-hypertensive and undiagnosed hypertensive group (76.65 and 0.97; 76.95 and 0.96 accordingly). Statistically significant associations of lower EQ-5D-5L indexes and EQ-VAS were found among people diagnosed for hypertension, people suffering from an incremental comorbidity, and obese people.

**Conclusion:**

This study has provided EQ-5D-5L population norms for the general population and evidence for known-groups validity of the EQ-5D-5L instrument among hypertensive people in Vietnam.

**Supplementary Information:**

The online version contains supplementary material available at 10.1007/s11136-021-02959-2.

## Introduction

High blood pressure is a silent killer associated with approximately 9.4 million cases of death a year worldwide [1]. The mortality rate from hypertensive heart disease has increased by 16.4% in the most recent decade in Vietnam [2]. The prevalence of Vietnamese aged 25–64 years living with hypertension has increased from 15.3 to 20.3% from 2010 to 2015, respectively [3]. The burden of the disease is spread out across socio-economic groups [4]. Vietnam’s Ministry of Health (MOH) is upgrading their system to provide sufficient healthcare services for people living with long-term illnesses, including hypertension [5]. Presently, the cost of more than 50 drugs for controlling high blood pressure have been completely covered by national health insurance in Viet Nam [6]. With a limited budget, the MOH is now applying evidence-based medicine to upgrade the national health insurance benefit package with cost-effective drugs [7]. Those facts imply a demand to promote evidence-informed policymaking in the national healthcare system, initially in health insurance. Nevertheless, efficient measures to evaluate the effectiveness of healthcare programmes/interventions are still in need [8]. Intermediate outcomes (e.g. levels of systolic/diastolic blood pressure—SBP|DBP) and natural measures (e.g. number of deaths or averted cases) are sometimes not adequate to reflect the effectiveness of interventions for the hypertensive population [9]. Hence, multi-dimensional health outcomes, such as the health-related quality of life (HRQOL), are now getting more attention for identifying additional health gains/losses offered by interventions on the hypertensive population [10]. Vietnam’s MOH has taken the first steps to make use of the HRQOL metric by requesting such outcomes and quality adjusted life years (QALY) in health technology assessments (HTA), especially pharma-economic [7]. These outcomes have also been requested by other countries when it comes to HTA [11–13]. In Vietnam, EQ-5D-5L is currently the only instrument that can produce the HRQOL metric that is based on preferences of the general Vietnamese population [14]. The EQ-5D-5L has also been suggested for the national HTA guidelines, yet there are still two big concerns regarding the instrument.

The first one is that Vietnam needs reference data allowing HRQOL comparisons between people with different characteristics (e.g. ages, sexes, illness status). The HRQOL reference data derived from EQ-5D-5L is normally referred to in name as “EQ-5D-5L population norms” [15]. The population norms typically provide three outcomes, including the reference data of descriptive five dimensional five levels and mean values of EQ-VAS, EQ-5D-5L indexes. The EQ-5D-5L population norms were developed globally, from Western countries [16–22] to Asian countries [23–29]. A Vietnamese population norms using the EQ-5D-5L has been done elsewhere, but the study included an urban population only, and furthermore, used Thai preferences [29]. Since Vietnam now has a country-specific value set [14], this is timely to develop the country-specific EQ-5D-5L population norms for the general population.

The second concern is whether EQ-5D-5L can be justified for use in Vietnam. Psychometric properties of the EQ-5D-5L have been proven in several countries and for several disease areas, including the instrument’s reliability and convergent and/or known-groups validation [30–37]. A study on the reliability and convergent validation of the EQ-5D-5L in Vietnam was conducted among HIV/AIDS patients [38], yet construction validation among people with hypertension is still limited. The approaches of construction validation for EQ-5D-5L commonly refer to convergent validation (estimating correlations of related dimensions of EQ-5D-5L and other instruments) [36, 37] and/or known-groups validation (evaluating the sensitiveness of the instrument by yielding distinct results among different groups of patients) [34, 36]. To fill the research gaps, this study aims to provide (1) HRQOL reference data using EQ-5D-5L among the general population and (2) construction validity tests for EQ-5D-5L instrument among people living with hypertension in Vietnam. Due to the shortage in HRQOL data measured by different instruments, only known-group validity tests were included in this article.

## Methodology

Data presented in this article was pooled from two separate studies. Data to derive the HRQOL reference data was taken from a Vietnam EQ-5D-5L valuation study which was conducted in the general population in 2017 [14]. The validity test was conducted using data from a survey from the “Evaluation of the Ho Chi Minh City Communities for Healthy Hearts” CH2 project, which was conducted in 2019 [39].

### Samples and data collection

All participants were recruited using the door-to-door approach. Interviewers at both studies had public health specialty and they carried out face-to-face interviews to collect the data at the households.

#### The HRQOL reference data

A general population sample of 1200 adults from the EQ-5D-5L valuation study [14] was selected to develop EQ-5D-5L population norms. A multi-stage, stratified, cluster probabilistic quota-based sampling method was applied. The first stage was to determine an urban and a rural cluster from six provinces of six different geographical regions. The next stage was to determine quotas for each cluster. The probabilistic quotas were developed based on the fractions of the population’s regions, residency, age groups (18–29 years old, 30–44 years old, 45–59 years old, and 60+ years old) and sex (male and female). Details of the sampling have been published elsewhere [14]. Data used for developing the population norms were participants’ demographic characteristics and self-reported health statuses using the EQ-5D-5L.

#### The validity testing of the EQ-5D-5L

A sample of 1296 adults aged 40 and above from the CH2 post-evaluation community survey [39] was used for the analysis of known-groups validity of the EQ-5D-5L. The sample was collected from eight districts of Ho Chi Minh City, the biggest megacity in the south of Vietnam. A combination of multistage, cluster, random sampling techniques was employed [39]. Data presented in this article included participants’ background, history on hypertension (physician diagnosed status and comorbidities), the EQ-5D-5L, and physical measurement outcomes (height, weight, blood pressure or BP). The trained interviewers brought along height-weight measures and BP monitors to households, and they conducted physical measurements of all participants after the interview [39]. All participants were asked to rest in the armchair for at least 15 min before the BP measurement and each participant had BP measurements taken twice on their left arm [39]. Average results of the BP measurements were reported in this study. Procedures concerning the physical measurements in the CH2 study adhered to the MOH’s guidelines on general health check-ups and physical measurements [40].

### Analysis

Generally, differences on distributions of the five dimensions, five levels among sub-groups were tested using Pearson Chi square tests. Due to the ceiling effect of the EQ-5D-5L instrument, non-parametric tests were used to test the differences of the EQ-VAS and EQ-5D-5L indexes among sub-groups, including Mann–Whitney tests for two-group categorical variables and Kruskal Wallis *H* Tests for more-than-two-group categorical variables. The Post-hoc analysis was performed to examine differences among multiple pairwise comparisons. A significance level of 0.05 was used for all statistical tests. Data was analysed using STATA version 17 software.

#### The EQ-5D-5L population norms

The EQ-5D-5L population norms were derived from the data given by the general population sample. The analysis on EQ-5D-5L population norms followed the standardised method recommended by the EuroQol Group [41]. Descriptive statistics of the five dimensions, five levels, EQ-VAS and EQ-5D-5L indexes, were categorised into gender and age groups. Among these, percentages of answers for the five dimensions, five levels were presented: EQ-VAS and EQ-5D-5L indexes were reported in means, standard deviations, ranges of min–max, and inter-quartiles, respectively. Differences of the EQ-5D-5L’ outcomes were statistically tested.

#### The known-groups validation

Literature suggested that the presence of hypertension and comorbidities was associated with lower HRQOL metrics [42–44]. Also, patients who were aware of their hypertensive status reported a poorer quality of life [45, 46]. Three groups were created based on the results of BP measurements and individual hypertension history. The first was a non-hypertensive group, which included 577 individuals who had never been diagnosed for hypertension by a physician and their average BP was below the World Health Organization's threshold for hypertension (meaning SBP|DBP ≤ 140|90 mmHg) [47]. The second was a diagnosed-hypertension group, including 477 hypertensive patients, meaning that they were once diagnosed for hypertension by physician(s) and were prescribed medication. According to the impact of medication, the average BP of individuals in this group was possibly at both below and above the hypertension threshold. The third was an un-diagnosed for hypertension group of 242 individuals who had never been diagnosed for hypertension by a physician, but their average BP (from physical measurements) was above the threshold for hypertension (meaning SBP|DBP ≥ 140|90 mmHg). Correspondingly, the known-group validation was tested, meaning, to see if EQ-VAS and EQ-5D-5L indexes would be higher among non-hypertensive people and among people with an undiagnosed hypertensive status than those of the hypertension-diagnosed group would. In addition, HRQOL of hypertensive people was often suggested to be lower among females, people with more comorbidities, less education, older ages, and higher body mass indexes (BMI) [48, 49]. Hence, these known-groups validations were tested for: gender, age, education, marital status, BMI classifications, and number of comorbidities. Associations of these known-groups with the EQ-VAS and EQ-5D-5L indexes were tested using a multivariate linear regression model.

## Results

**Fig. 1:**
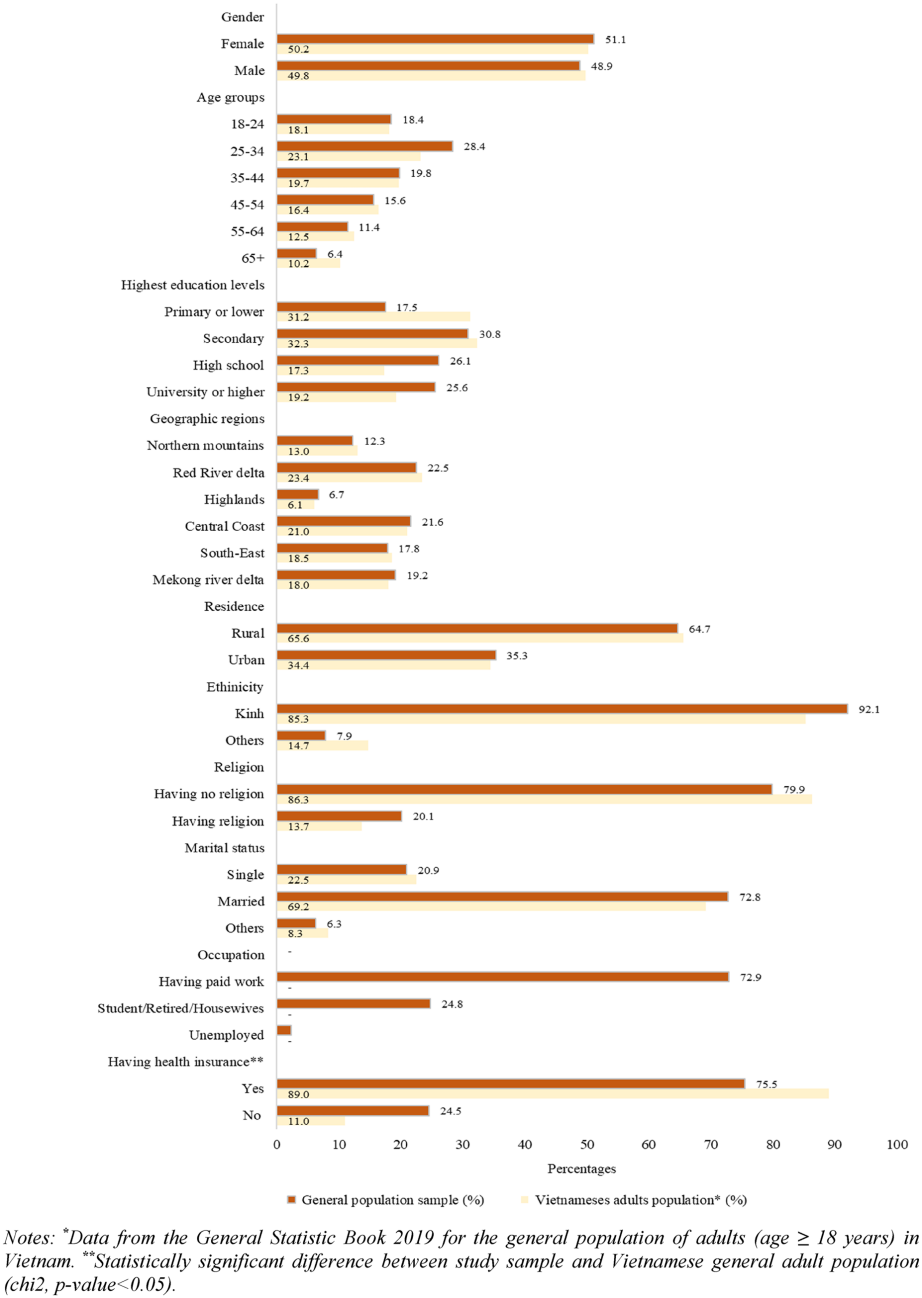
Pattern of the general population sample and Vietnamese general adult population

Figure 1 shows characteristics of the general population sample. Overall, the sample distribution was similar among sub-groups of genders, age groups, geographic regions, and education levels. Socio-demographic characteristics of the present sample were in line with those of the national adult population. Nevertheless, this sample included younger and highly educated people than the national average.

Generally, the percentage of participants that reported having full health was 54.4% and such indexes decreased by age (Table 1). Respondents reported fewer problems in self-care and usual activities than the other dimensions. The number of individuals that reported having problems at higher levels increased for the subsequent age groups. Females reported to have more problems in mobility, pain/discomfort, anxiety/depression than males in all age groups. Details of the five dimensions, five levels for females-males in urban–rural subgrouping by age are presented in Table 1a|b in online resource.

**Table 1:** Percentage of a general population sample reporting the five dimensions by age groups

		18–24	25–34	35–44	45–54	55–64	65 +	Total
		*n* = *220*	*n* = *342*	*n* = *237*	*n* = *187*	*n* = *137*	*n* = *77*	*n* = 1*200*
Mobility	No problems	98.2%	95.9%	93.2%	83.4%	81.0%	68.8%	90.4%
	Slight problems	1.8%	4.1%	5.5%	15.0%	16.8%	26.0%	8.5%
	Moderate problems			0.4%	0.5%	1.5%	2.6%	0.5%
	Severe problems			0.8%	1.1%	0.7%	2.6%	0.6%
	Unable to walk							
	Pearson chi^2^ (Pr)*	100.9 (**< 0.01**)						
Self-care	No problems	100%	99.4%	98.7%	97.9%	94.9%	94.8%	98.3%
	Slight problems		0.6%	0.4%	2.1%	5.1%	3.9%	1.4%
	Moderate problems			0.8%			1.3%	0.3%
	Severe problems							
	Unable to wash or dress							
	Pearson chi2 (Pr)*	32.9 (**< 0.01**)						
Usual activities	No problems	98.6%	96.2%	95.3%	95.2%	92.0%	88.3%	95.3%
	Slight problems	1.4%	3.8%	3.0%	4.8%	8.0%	10.4%	4.3%
	Moderate problems			1.7%				0.3%
	Severe problems						1.3%	0.1%
	Unable to do usual activities							
	Pearson chi2 (Pr)*	48.7 (**< 0.01**)						
Pain/discomfort	No pain	76.4%	78.0%	65.4%	49.2%	52.6%	45.4%	65.7%
	Slight pain	21.3%	19.9%	31.2%	41.7%	39.4%	39.0%	29.3%
	Moderate pain	2.3%	2.1%	2.1%	6.4%	5.1%	15.6%	4.0%
	Severe pain			1.3%	2.7%	2.9%		1.0%
	Extreme pain							
	Pearson chi2 (Pr)*	113.2 (**< 0.01**)						
Anxiety/depression	Not anxious	76.4%	83.5%	80.2%	82.9%	75.9%	80.5%	80.3%
	Slightly anxious	20.4%	15.0%	15.6%	13.4%	19.0%	14.3%	16.3%
	Moderately anxious	3.2%	0.6%	2.5%	2.1%	2.9%	5.2%	2.3%
	Severely anxious		0.6%	1.3%	1.6%	2.2%	0.0%	0.9%
	Extremely anxious		0.3%	0.4%	0.0%	0.0%	0.0%	0.2%
	Pearson chi2 (Pr)*	23.3 (0.27)						
Reporting full health		61.1%	64.2%	53.6%	42.2%	46.7%	37.7%	54.4%
	Pearson chi2 (Pr)*	40.4 (**< 0.01**)						

Bold values denote statistical significance at the *p* < 0.05 level

*n* number of individuals

*Results from Pearson Chi square Tests.

### The EQ-5D-5L population norms

Overall, the mean EQ-VAS and EQ-5D-5L indexes were 81.10 (SD: 13.35) and 0.94 (SD: 0.09) (Table 2), respectively. The mean EQ-VAS and EQ-5D-5L indexes were statistically significantly higher among younger people, males, people having an education level at high school or higher, or those not being unemployed, or single (*p* value ≤ 0.05). By geographical region, the EQ-VAS was shown to be statistically lower among people living in the Central Coast areas. Results of EQ-VAS, EQ-5D-5L indexes among females-males in urban–rural subgrouping by age are presented in Table 2a|b and the Post-hoc analysis is presented in Table 2c|d in online resource.

**Table 2:** EQ VAS, EQ−5D−5L indexes among the Vietnamese general population

	EQ VAS			EQ-5D-5L values		
	Mean (SD)	Min–Max; IQR	*p* value	Mean (SD)	Min–Max; IQR	*p* value
Total	81.10 (13.35)	10–100; 20		0.94 (0.09)	0.29–1; 0.08	
Gender*						
Female	80.38 (13.70)	10–100; 20	**0.05**	0.93 (0.09)	0.29–1; 0.08	** < 0.01**
Male	81.84 (12.94)	30–100; 11		0.95 (0.08)	0.36–1; 0.08	
Age group**						
18–24	83.96 (10.26)	50–100; 10	** < 0.01**	0.96 (0.06)	0.76–1; 0.08	** < 0.01**
25–34	84.36 (11.04)	40–100; 10		0.96 (0.06)	0.68–1; 0.07	
35–44	81.75 (13.80)	20–100; 15		0.94 (0.09)	0.29–1; 0.08	
45–54	78.73 (13.69)	50–100; 20		0.92 (0.1)	0.56–1; 0.15	
55–64	74.82 (15.93)	10–100; 15		0.91 (0.11)	0.49–1; 0.15	
65+	73.36 (15.44)	40–100; 10		0.89 (0.12)	0.42–1; 0.15	
Highest education**						
Primary and lower	77.67 (16.10)	20–100; 20	** < 0.01**	0.92 (0.12)	0.29–1; 0.08	** < 0.01**
Secondary	79.84 (14.06)	10–100; 20		0.93 (0.09)	0.49–1; 0.08	
High school	82.56 (11.59)	50–100; 10		0.95 (0.07)	0.62–1; 0.08	
Undergraduate and higher	83.43 (11.34)	40–100; 10		0.95 (0.06)	0.57–1; 0.08	
Geographic regions**						
Northern mountains	80.10 (13.01)	35–100; 20	** < 0.01**	0.93 (0.10)	0.36–1; 0.13	0.26
Red River delta	82.05 (11.79)	40–100; 11		0.95 (0.07)	0.67–1; 0.08	
Highlands	80.06 (13.08)	50–100; 20		0.94 (0.09)	0.49–1; 0.08	
Central Coast	77.80 (15.35)	20–100; 20		0.93 (0.11)	0.29–1; 0.08	
South-East	82.43 (11.89)	50–100; 10		0.95 (0.07)	0.57–1; 0.08	
Mekong river delta	83.43 (13.56)	10–100; 10		0.95 (0.08)	0.56–1; 0.08	
Residence*						
Rural	80.70 (13.63)	20–100; 20	0.25	0.94 (0.09)	0.29–1; 0.08	0.78
Urban	81.80 (12.82)	10–100; 15		0.94 (0.08)	0.49–1; 0.08	
Ethnicity*						
Kinh (as majority)	81.24 (13.39)	10–100; 20	0.10	0.94 (0.08)	0.29–1; 0.08	**0.01**
Others	79.34 (12.86)	40–100; 20		0.92 (0.11)	0.36–1; 0.15	
Religion*						
Having no religion	81.34 (13.23)	10–100; 20	0.24	0.94 (0.09)	0.29–1; 0.08	0.75
Having a religion	80.11 (13.81)	30–100; 20		0.94 (0.09)	0.56–1; 0.08	
Marital status**						
Single	83.47 (11.28)	50–100; 10	** < 0.01**	0.96 (0.06)	0.68–1; 0.08	** < 0.01**
Married	80.70 (13.69)	10–100; 20		0.94 (0.09)	0.29–1; 0.08	
Separated/widowed/divorced	77.68 (14.70)	40–100; 20		0.91 (0.11)	0.42–1; 0.15	
Occupation**						
Having paid work	81.85 (12.88)	20–100; 15	** < 0.01**	0.95 (0.08)	0.29–1; 0.08	0.06
Student/retired/housewives	79.93 (14.06)	10–100; 20		0.93 (0.09)	0.56–1; 0.08	
Unemployed	69.26 (14.46)	40–90; 20		0.89 (0.16)	0.42–1; 0.15	
Having health insurance*						
No	80.41 (13.72)	30–100; 20	0.34	0.95 (0.08)	0.57–1; 0.08	**0.01**
Yes	81.31 (13.23)	10–100; 20		0.94 (0.09)	0.29–1; 0.08	

Bold values denote statistical significance at the *p* < 0.05 level

*Results from Mann–Whitney tests; **Results from Kruskal Wallis H-Tests *SD* Standard Deviation, *IQR* interquartile range

### Known-groups validation

The sample of the diagnosed hypertensive group was older and had more comorbidities than the non-hypertensive, non-diagnosed hypertensive group (Table 3). The smallest percentage of “full health” was 62.68% among the diagnosed group. The mean EQ-VAS and EQ-5D-5L indexes of people in the diagnosed hypertensive group (71.48 and 0.94, respectively) were statistically significantly smaller than they were in the other two groups (*p* value < 0.05). Results of EQ-VAS and EQ-5D-5L indexes were reported comparably between people from the non-hypertensive and undiagnosed hypertensive group (76.65 and 0.97; 76.95 and 0.96 accordingly). Results of the Post-hoc analysis for CH2 sample are presented in Table 3a|b, Online resource.

**Table 3:** EQ VAS, EQ−5D−5L indexes across hypertensive groups

	Non-hypertensive group			Diagnosed with hypertension group			Un-diagnosed with hypertension group		
	*n* (%)	VAS (mean; SD)	Value (mean; SD)	*n* (%)	VAS (mean; SD)	Value (mean; SD)	*n* (%)	VAS (mean; SD)	Value (mean; SD)
***N***	**577**			**477**			**242**		
Reporting full health	415 (71.92)			299 (62.68)			174 (71.90)		
Age group									
40–49	268 (46.45)	77.39 (14.43)	0.97 (0.06)	88 (18.45)	75.52 (14.63)	0.95 (0.11)	92 (38.02)	75.48 (13.65)	0.97 (0.07)
50–59	200 (34.66)	76.86 (14.47)	0.96 (0.07)	164 (34.38)	72.07 (17.55)	0.94 (0.11)	90 (37.19)	77.91 (12.93)	0.96 (0.1)
60+	109 (18.89)	74.45 (14.39)	0.97 (0.05)	225 (47.17)	69.51 (16.11)	0.93 (0.14)	60 (24.79)	77.76 (17.03)	0.97 (0.05)
*p* value**		0.11	0.08		** < 0.01**	0.43		0.29	0.98
Gender									
Female	341 (59.10)	77.12 (14.96)	0.96 (0.07)	341 (59.10)	70.73 (16.75)	0.92 (0.14)	70 (28.93)	77.9 (13.32)	0.95 (0.1)
Male	236 (40.90)	75.97 (13.69)	0.98 (0.05)	236 (40.90)	72.25 (16.21)	0.95 (0.11)	172 (71.07)	76.55 (14.7)	0.97 (0.07)
*p* value*		0.22	**0.02**		0.31	**0.01**		0.69	**0.13**
Marital status									
Single	30 (5.20)	75.97 (14.1)	0.96 (0.06)	22 (4.61)	69 (26.66)	0.93 (0.12)	10 (4.13)	80.8 (15.87)	0.96 (0.06)
Married	467 (80.94)	76.72 (14.16)	0.97 (0.06)	373 (78.20)	72.19 (15.71)	0.94 (0.13)	207 (85.54)	77.05 (14.42)	0.97 (0.06)
Separated/divorce/widow	80 (13.86)	76.5 (16.33)	0.96 (0.08)	82 (17.19)	68.96 (16.34)	0.93 (0.09)	25 (10.33)	74.6 (12.74)	0.91 (0.16)
* p* value**		0.79	0.90		0.22	0.34		0.48	0.06
Highest education									
Primary and lower	246 (42.63)	76.09 (16.25)	0.96 (0.07)	210 (44.03)	69.72 (16.76)	0.92 (0.14)	106 (43.80)	78.06 (15.08)	0.97 (0.09)
Secondary	167 (28.94)	76.08 (12.77)	0.97 (0.05)	139 (29.14)	72.07 (17.18)	0.95 (0.09)	70 (28.93)	75.58 (12.73)	0.97 (0.06)
High school and higher	164 (28.42)	78.07 (13.13)	0.98 (0.05)	128 (26.83)	73.72 (15.01)	0.95 (0.12)	66 (27.27)	76.63 (14.63)	0.96 (0.07)
*p* value**		0.45	0.12		**0.05**	0.24		0.25	0.44
Body Mass Index									
< 18.5 (underweight)	51 (8.84)	71.08 (14.88)	0.96 (0.06)	26 (5.45)	68.27 (14.14)	0.95 (0.1)	13 (5.37)	74.62 (13.46)	0.98 (0.04)
18.5–24.9 (normal)	421 (72.96)	76.79 (14.36)	0.97 (0.06)	293 (61.43)	71.63 (16.79)	0.94 (0.11)	166 (68.60)	77.02 (15.08)	0.97 (0.06)
< 25 (overweight/ obesity)	105 (18.20)	78.81 (14.08)	0.97 (0.06)	158 (33.12)	71.74 (16.31)	0.93 (0.15)	63 (26.03)	77.26 (12.37)	0.95 (0.11)
*p* value**		** < 0.01**	0.41		0.58	0.94		0.97	0.91
Occupation									
Unemployed	29 (5.03)	73.62 (15.11)	0.97 (0.05)	70 (14.68)	68.86 (18.96)	0.89 (0.18)	30 (12.40)	72.76 (16.23)	0.95 (0.10)
Having a paid job	548 (94.97)	76.81 (14.41)	0.97 (0.06)	407 (85.32)	71.94 (16.00)	0.94 (0.11)	212 (87.60)	77.53 (13.95)	0.97 (0.07)
*p* value*		0.26	0.80		0.18	0.04		0.15	0.77
Having comorbidity									
No	510 (88.39)	77.05 (14.28)	0.97 (0.06)	311 (65.20)	72.58 (15.36)	0.95 (0.11)	219 (90.50)	77.72 (13.58)	0.97 (0.08)
Yes	67 (11.61)	73.66 (15.51)	0.95 (0.07)	166 (34.80)	69.44 (18.27)	0.91 (0.15)	23 (9.50)	69.78 (18.68)	0.96 (0.09)
*p* value*		0.07	** < 0.01**		0.06	** < 0.01**		**0.03**	**0.76**
Hypertension status	EQ-VAS (mean; SD)				EQ-5D-5L index (mean; SD)				
Non-hypertensive	76.65 (14.45)				0.97 (0.06)				
Un-diagnosed for hypertension	76.95 (14.29)				0.96 (0.08)				
Diagnosed for hypertension	71.48 (16.48)				0.94 (0.12)				
*p* value**	** < 0.01**				** < 0.01**				

Bold values denote statistical significance at the *p* < 0.05 level

*Results from Mann–Whitney tests; **Results from Kruskal Wallis *H* Tests

*SD* Standard Deviation

Results show statistically significant decreases in the EQ-VAS among people diagnosed for hypertension, higher numbers of comorbidities, and those underweight (Table 4). Statistically significant associations of lower EQ-5D-5L indexes were found among people diagnosed for hypertension, people of older ages, females, people suffering from an incremental comorbidity, and obese people. People with higher education levels may associate with higher both EQ-VAS and EQ-5D-5L indexes.

**Table 4:** Factors associate with the EQ-VAS and EQ-5D-5L indexes

	EQ VAS			EQ-5D-5L index		
	Coeff.	Std. Err.	95% CI	Coeff.	Std. Err.	95% CI
Hypertensive group (Ref: Non-hypertensive)						
Diagnosed with hypertension group	− 3.731	1.035	**[− 5.76; − 1.701]**	**− **0.022	0.006	**[− 0.034; -0.009]**
Un-diagnosed with hypertension group	0.143	1.197	[**− **2.206; 2.491]	**− **0.006	0.007	[**− **0.021; 0.008]
Age	**− **0.076	0.046	[**− **0.167; 0.015]	**− **0.001	0.000	**[− 0.001; 0]**
Gender (Ref: Male)						
Female	0.082	0.898	[**− **1.68; 1.844]	**− **0.017	0.005	**[− 0.028; -0.007]**
Education (Ref: primary school)						
Secondary school	0.117	1.012	[**− **1.869; 2.102]	0.015	0.006	**[0.003; 0.027]**
High school and higher	1.839	1.032	[**− **0.186; 3.863]	0.014	0.006	**[0.002; 0.026]**
Relationship (Ref: Doesn't have a partner)						
Has a partner	0.985	1.119	[**− **1.21; 3.18]	0.002	0.007	[**− **0.012; 0.015]
Number of comorbidities	**− **2.678	0.599	**[− 3.852; − 1.503]**	**− **0.013	0.004	**[− 0.02; -0.006]**
BMI (Ref: Normal from 18.5 to 24.9)						
Underweight (BMI < 18.5)	**− **4.164	1.698	**[− 7.494; − 0.833]**	0.000	0.010	[**− **0.02; 0.02]
Overweight or Obesity (BMI > 25)	0.928	0.998	[**− **1.03; 2.887]	-0.009	0.006	[**− **0.021; 0.003]
Constant	79.850	2.945	[74.073; 85.628]	1.007	0.018	[0.97; 1.04]
*R*^2^	0.056			0.059		
* n*	1285			1296		

Bolded 95% CI represents statistically significant differences in sub-groups

*Coeff* Coefficient, *Std.Err.* Standard error. *95% CI* 95% confident interval, *BMI* Body Mass Index, *n* number of individuals

## Discussion

This study has provided EQ-5D-5L reference data in Vietnam, which was presented with regards to age and gender for the descriptive part of the five dimensions, five levels, EQ-VAS and EQ-5D-5L indexes. Additionally, this study demonstrated the validity of the EQ-5D-5L instrument among people living with hypertension. EQ-5D-5L was shown to be possible to capture changes in HRQOLs among participants with less desirable health statuses.

A strength of this study’s EQ-5D-5L population norms was the neutral context sample. Responses were pooled across the country by geographical regions, gender, age, and residence settings. In a previous EQ-5D-5L population norms study, results were derived from the data of an urban population and EQ-5D-5L indexes were calculated using a Thai value set [29]. However, EQ-5D-5L indexes in the present study were estimated using the Vietnamese preference-based value set. In addition, the percentage reporting full health in the previous study was about 67.4%, which was 13% higher than the present study. Findings here of EQ-5D-5L population norms, therefore, could be perceived as more neutral context HRQOL reference data. The mean EQ-5D-5L value for Vietnamese adults was about 0.94, which was in line with the range of indexes across countries, from 0.89 in Poland [16] to 0.96 in China [23]. Patterns of the EQ-5D-5L reference data found in the present study were similar with a previous Vietnamese EQ-5D-5L population norms study [29], China [23], Hong Kong [25], Indonesia [26] and Spain [19]; for example, the EQ-5D-5L indexes were reported to be lower for females than males, or higher for people having an education from high school and higher. The EQ-5D-5L indexes in this study showed a linear relationship with age for both genders. Nevertheless, the linear relationship was inconsistent for females, i.e., the mean EQ-5D-5L value was slightly lower among younger females aged 18-24 years than those in the age group of 25-34 years. EQ-5D-5L population norms in Australia [18] and Hong Kong [25] also reported similar linear relationships between the EQ-5D-5L value and age. Moreover, results showed statistically significant differences of EQ-VAS across the six geographical regions, where seemingly people from mountainous or poorer regions (e.g., the Central coast, the Highlands, the northern mountainous areas) were more likely to have lower EQ-VAS and EQ-5D-5L indexes than the others. This may have implied a hint of inequity in people’s HRQOL overall, in examples regarding education status, residential areas, occupation, marital status, ethnicity, and more.

With respect to the known-groups validation, the EQ-5D-5L performed in such a way that better HRQOL was more frequently reported by both people living with better health and people not being aware of their disease. The rates of people at full health, the EQ-VAS and EQ-5D-5L indexes, were similar between the two groups of non-hypertensive and undiagnosed hypertensive individuals, while such indicators were lower among those with diagnosed hypertension. Similar findings were also found in a study in Rio [50]. The presence of a clinical diagnosis for hypertension was statistically associated with 0.03 lower EQ-5D-5L indexes and 5 points lower for the EQ-VAS, which was in line with a similar study in China [49]. Moreover, the EQ-VAS and the EQ-5D-5L indexes were proven to be higher among people who did not have any comorbidity, as in previous literature [43, 51]. Generally, the known-groups validity of the EQ-5D-5L instrument has been verified among Vietnamese living with hypertension. Whilst higher EQ-5D-5L indexes were associated with people who have completed college or higher, the lower EQ-5D-5L indexes were associated with older age, being female, having BMI classified as obese, and having more comorbidities among people diagnosed for hypertension. Such findings aligned with both international [42–45] and national [51–53] literature.

With respect to the use of EQ-5D-5L population norms as a reference to compare HRQOL, the results found in this study appear to suggest that people with hypertension may have lower EQ-VAS points, but higher EQ-5D-5L indexes than the general population. EQ-VAS among diagnosed and undiagnosed groups for hypertension were at 71.48 and 76.96, whereas the EQ-VAS among the general population at the same age (40 years and above) ranged from 73.36 to 78.73. For the EQ-5D-5L indexes, the range of the general population was from 0.89 to 0.92, whilst it was 0.96 among the hypertension-undiagnosed group, and 0.94 among the diagnosed group. The average HRQOL of non-hypertensive people from the CH2 project was also reported to be higher than that of the general population. However, the higher HRQOL of people from the CH2 cohort in comparison with the general population can be explained by the better living conditions of the CH2 population, as 95% of participants from the CH2 project had a paid job, and their residence had received investment from the Head of the Ho Chi Minh City’s People Committee with several urban infrastructure and healthcare interventions.

Several limitations are found in this study. First, the sample for implementing the validation test was not contextually neutral. When the EQ-5D-5L population norms sample included all six country regions and residence types, the validation tests were taken from a study conducted in a megacity in South Vietnam and including only an urban population. In addition, the lack of HRQOL measurements from different instruments and at different time points limited the ability to test the reliability and sensitivity of the EQ-5D-5L instrument. Hence, this present study touched only a trivial part of the psychometric properties of the EQ-5D-5L.

## Conclusion

This study has provided EQ-5D-5L population norms for the general population and evidence for known-groups validity of the EQ-5D-5L instrument among hypertensive people in Vietnam. Findings from this study have addressed two main literature gaps in Vietnam, which were: (1) the population norms in context neutral HRQOL reference data, and (2) the known-groups validity of the EQ-5D-5L having been tested among people with different statuses of hypertension.

## Supplementary Information

Below is the link to the electronic supplementary material.

Supplementary file1 (PDF 296 kb)

Supplementary file2 (XLSX 210 kb)

## References

[1] A comparative risk assessment of burden of disease and injury attributable to 67 risk factors and risk factor clusters in 21 regions, 1990–2010: a systematic analysis for the Global Burden of Disease Study 2010 - The Lancet. (n.d.). Retrieved May 21, 2021, from https://www.thelancet.com/journals/lancet/article/PIIS0140-6736(12)61766-8/fulltext 10.1016/S0140-6736(12)61766-8 PMC4156511 23245609

[2] Vos, T., Lim, S. S., Abbafati, C., Abbas, K. M., Abbasi, M., Abbasifard, M., … Murray, C. J. L. (2020). Global burden of 369 diseases and injuries in 204 countries and territories, 1990–2019: a systematic analysis for the Global Burden of Disease Study 2019. *The Lancet*, *396*(10258), 1204–122210.1016/S0140-6736(20)30925-9 10.1016/S0140-6736(20)30925-9 PMC7567026 33069326

[3] Ministry of Health, General Department of Preventive Medicine. (2015). Global burden of 369 diseases and injuries in 204 countries and territories, 1990–2019: a systematic *National survey on the risk factors of non-communicable diseases (STEPS) Viet Nam, 2015*.

[4] Hoang, V. M., Tran, Q. B., Vu, T. H. L., Nguyen, T. K. N., Kim, B. G., Pham, Q. N., … Tran, D. P. (2019). Patterns of Raised Blood Pressure in Vietnam: Findings from the WHO STEPS Survey 2015. *International Journal of Hypertension*. Research Article. 10.1155/2019/1219783 10.1155/2019/1219783 PMC6913158 31871783

[5] Vietnam Ministry of Health, Health Partnership Group,. (2015). *Joint Annual Health Review Report - 2015: Strengthening primary health care at the grassroots towards universal health coverage*. Vietnam.

[6] Ministry of Health. Circular 30/2018/TT-BYT on Promulgation of list of modern medicines, biologicals, radiopharmaceuticals and tracers covered by health insurance, insurance coverage ratio and payment conditions thereof. , 30/2018/TT-BYT 30/2018/TT-BYT § Annex 1.

[7] Ministry of Health. Decision 5315/QD-BYT on the principles and criteria for the formulation of list of new drugs under the national health insurance scheme, Code 5315/QD-BYT/2018 (enacted date: 31/08/2018).

[8] Vietnam Ministry of Health, Health Partnership Group,. (2014). *Joint Annual Health Review Report - 2014: Strengthening prevention and control of non-communicable disease*.

[9] Drummond MF (1987). Resource allocation decisions in health care: A role for quality of life assessments?. Journal of Chronic Diseases.

[10] Drummond MF, Sculpher MJ, Claxton K, Stoddart GL, Torrance GW (2015). Methods for the Economic Evaluation of Health Care Programmes.

[11] NICE. (2013). Guide to the methods of technology appraisal 2013 in UK. Retrieved February 4, 2020, from https://www.nice.org.uk/process/pmg9/chapter/foreword

[12] CADTH,. (2015, December 2). Guidelines for the Economic Evaluation of Health Technologies: Canada. *CADTH.ca*. Retrieved February 4, 2020, from https://www.cadth.ca/about-cadth/how-we-do-it/methods-and-guidelines/guidelines-for-the-economic-evaluation-of-health-technologies-canada

[13] Teerawattananon, Y., & Chaikledkaew, U. (2008). Thai health technology assessment guideline development. *Journal of the Medical Association of Thailand = Chotmaihet Thangphaet*, *91 Suppl 2*, S11–15. 19253483

[14] Mai VQ, Sun S, Minh HV, Luo N, Giang KB, Lindholm L, Sahlen KG (2020). An EQ-5D-5L value set for vietnam. Quality of Life Research.

[15] Janssen B, Szende A, Szende A, Janssen B, Cabases J (2014). Population norms for the EQ-5D. Self-reported population health: An International Perspective based on EQ-5D.

[16] Golicki D, Niewada M (2017). EQ-5D-5L Polish population norms. Archives of Medical Science : AMS.

[17] Bailey H, Janssen MF, La Foucade A, Kind P (2019). EQ-5D-5L population norms and health inequalities for Trinidad and Tobago. PLoS ONE.

[18] McCaffrey N, Kaambwa B, Currow DC, Ratcliffe J (2016). Health-related quality of life measured using the EQ-5D-5L: South Australian population norms. Health and Quality of Life Outcomes.

[19] Garcia-Gordillo MA, Adsuar JC, Olivares PR (2016). Normative values of EQ-5D-5L: In a Spanish representative population sample from Spanish Health Survey, 2011. Quality of Life Research: An International Journal of Quality of Life Aspects of Treatment, Care and Rehabilitation.

[20] Encheva M, Djambazov S, Vekov T, Golicki D (2020). EQ-5D-5L Bulgarian population norms. The European journal of health economics: HEPAC: Health economics in prevention and care.

[21] Health Quality Council of Alberta. (2016). *EQ-5D-5L index norms for Alberta population*. Retrieved from https://www.hqca.ca/wp-content/uploads/2018/05/HQCA_EQ_5D_AB_Norms_Report_Addendum_DRAFT___June_28.pdf

[22] Prevolnik Rupel V, Ogorevc M (2020). EQ-5D-5L Slovenian population norms. Health and Quality of Life Outcomes.

[23] Yang Z, Busschbach J, Liu G, Luo N (2018). EQ-5D-5L norms for the urban Chinese population in China. Health and Quality of Life Outcomes.

[24] Shiroiwa T, Fukuda T, Ikeda S, Igarashi A, Noto S, Saito S, Shimozuma K (2016). Japanese population norms for preference-based measures: EQ-5D-3L, EQ-5D-5L, and SF-6D. Quality of Life Research: An International Journal of Quality of Life Aspects of Treatment, Care and Rehabilitation.

[25] Wong EL-Y, Cheung AW-L, Wong AY-K, Xu RH, Ramos-Goñi JM, Rivero-Arias O (2019). Normative profile of health-related quality of life for Hong Kong general population using preference-based instrument EQ-5D-5L. Value in Health: The Journal of the International Society for Pharmacoeconomics and Outcomes Research.

[26] Purba FD, Hunfeld JAM, Iskandarsyah A, Fitriana TS, Sadarjoen SS, Passchier J, Busschbach JJV (2018). Quality of life of the Indonesian general population: Test-retest reliability and population norms of the EQ-5D-5L and WHOQOL-BREF. PLoS ONE.

[27] Emrani Z, Akbari Sari A, Zeraati H, Olyaeemanesh A, Daroudi R (2020). Health-related quality of life measured using the EQ-5D-5L: Population norms for the capital of Iran. Health and Quality of Life Outcomes.

[28] Vaingankar, J. A., Subramaniam, M., Tan, L. W. L., Abdin, E., Lim, W. Y., Wee, H. L., … van Dam, R. M. (2018). Psychometric properties and population norms of the positive mental health instrument in a representative multi-ethnic Asian population. *BMC Medical Research Methodology*, *18*(1), 2910.1186/s12874-018-0487-9 10.1186/s12874-018-0487-9 PMC5856373 29544448

[29] Nguyen LH, Tran BX, Le Hoang Q, N., Tran, T. T., & Latkin, C. A. (2017). Quality of life profile of general Vietnamese population using EQ-5D-5L. Health and Quality of Life Outcomes.

[30] Rencz, F., Lakatos, P. L., Gulácsi, L., Brodszky, V., Kürti, Z., Lovas, S., … Palatka, K. (2019). Validity of the EQ-5D-5L and EQ-5D-3L in patients with Crohn’s disease. *Quality of Life Research: An International Journal of Quality of Life Aspects of Treatment, Care and Rehabilitation*, *28*(1), 141–15210.1007/s11136-018-2003-4 10.1007/s11136-018-2003-4 30225788

[31] Nolan CM, Longworth L, Lord J, Canavan JL, Jones SE, Kon SSC, Man WD-C (2016). The EQ-5D-5L health status questionnaire in COPD: Validity, responsiveness and minimum important difference. Thorax.

[32] Cheung PWH, Wong CKH, Samartzis D, Luk KDK, Lam CLK, Cheung KMC, Cheung JPY (2016). Psychometric validation of the EuroQoL 5-Dimension 5-Level (EQ-5D-5L) in Chinese patients with adolescent idiopathic scoliosis. Scoliosis and Spinal Disorders.

[33] Bilbao, A., García-Pérez, L., Arenaza, J. C., García, I., Ariza-Cardiel, G., Trujillo-Martín, E., & Martín-Fernández, J. (2018). Psychometric properties of the EQ-5D-5L in patients with hip or knee osteoarthritis: Reliability, validity and responsiveness. *Quality of Life Research: An International Journal of Quality of Life Aspects of Treatment, Care and Rehabilitation*, *27*(11), 2897–290810.1007/s11136-018-1929-x 10.1007/s11136-018-1929-x 29978346

[34] Koh D, Abdullah AMKB, Wang P, Lin N, Luo N (2016). Validation of Brunei’s Malay EQ-5D questionnaire in patients with Type 2 diabetes. PLoS ONE.

[35] Purba FD, Hunfeld JAM, Buczek R, Timman A, Iskandarsyah A, Fitriana TS, Sadarjoen SS, Passchier J, Busschbach JJV (2015). Test-retest reliability of EQ-5D-5L valuation techniques: the composite time trade-off and discrete choice experiments. Value in Health.

[36] Golicki D, Niewada M, Buczek J, Karliñska, A, Kobayashi A, Janssen MF, Pickard AS (2015). Validity of EQ-5D-5L in stroke. Quality of Life Research: An International Journal of Quality of Life Aspects of Treatment, Care and Rehabilitation.

[37] Sakthong P, Sonsa-Ardjit N, Sukarnjanaset P, Munpan W (2015). Psychometric properties of the EQ-5D-5L in Thai patients with chronic diseases. Quality of Life Research: An International Journal of Quality of Life Aspects of Treatment, Care and Rehabilitation.

[38] Tran BX, Ohinmaa A, Nguyen LT (2012). Quality of life profile and psychometric properties of the EQ-5D-5L in HIV/AIDS patients. Health and Quality of Life Outcomes.

[39] Nguyen, T. N. P., Hoang, V. M., Tran, T. N., Shellaby, J. T., Adler, A. J., McGuire, H., … Do, V. V. (2020). Knowledge change related to hypertension in the Southern province of Vietnam: a community based, before and after intervention evaluation. *Journal of Global Health Science*, *2*(1), e14. 10.35500/jghs.2020.2.e14

[40] Ministry of Health. National guidline for commune health center in diagnosis and treatment of Hypertension. , No 3192/QD-BYT No 3192/QD-BYT (2010). Retrieved from https://kcb.vn/wp-content/uploads/2015/07/huong_dan_chan_doan_dieu_tri_tha.pdf

[41] Janssen B, Szende A, Ramos-Goñi JM, Szende A, Janssen B, Cabases J (2014). Data and methods. Self-reported population health: An International Perspective based on EQ-5D.

[42] Ye R, Liu K, Zhang Z, Gong S, Chen X (2018). Health-related quality of life of hypertension in China: A systematic review and meta-analysis. Journal of Cardiovascular Medicine.

[43] Bao X-Y, Xie Y-X, Zhang X-X, Peng X, Huang J-X, Du Q-F, Wang P-X (2019). The association between multimorbidity and health-related quality of life: A cross-sectional survey among community middle-aged and elderly residents in southern China. Health and Quality of Life Outcomes.

[44] Khalifeh M, Salameh P, Hajje AA, Awada S, Rachidi S, Bawab W (2015). Hypertension in the Lebanese adults: Impact on health related quality of life. Journal of Epidemiology and Global Health.

[45] Korhonen PE, Kivelä S-L, Kautiainen H, Järvenpää S, Kantola I (2011). Health-related quality of life and awareness of hypertension. Journal of Hypertension.

[46] Mi B, Dang S, Li Q, Zhao Y, Yang R, Wang D, Yan H (2015). Association between awareness of hypertension and health-related quality of life in a cross-sectional population-based study in rural area of Northwest China. Medicine.

[47] World Health Organization. (n.d.). *A global brief on hypertension: Silent killer, global public health crisis* (No. WHO/DCO/WHD/2013/2). Retrieved from https://apps.who.int/iris/handle/10665/79059

[48] Riley E, Chang J, Park C, Kim S, Song I (2019). Hypertension and Health-Related Quality of Life (HRQoL): Evidence from the US Hispanic Population. Clinical Drug Investigation.

[49] Zhang L, Guo X, Zhang J, Chen X, Zhou C, Ge D, Qian Y (2017). Health-related quality of life among adults with and without hypertension: A population-based survey using EQ-5D in Shandong China. Scientific Reports.

[50] Mena-Martin FJ, Martin-Escudero JC, Simal-Blanco F, Carretero-Ares JL, Arzua-Mouronte D, Herreros-Fernandez V (2003). Health-related quality of life of subjects with known and unknown hypertension: Results from the population-based Hortega study. Journal of Hypertension.

[51] Vu, H. M., Nguyen, L. H., Tran, T. H., Pham, K. T. H., Phan, H. T., Nguyen, H. N., … Ho, R. C. M. (2019). Effects of Chronic Comorbidities on the Health-Related Quality of Life among Older Patients after Falls in *Vietnamese Hospitals. *International Journal of Environmental Research and Public Health*, *16*(19). 10.3390/ijerph16193623 10.3390/ijerph16193623 PMC6801440 31569612

[52] Nguyen T-P-L, Krabbe PFM, Nguyen T-B-Y, Schuiling-Veninga CCM, Wright EP, Postma MJ (2015). Utilities of Patients with Hypertension in Northern Vietnam. PLoS ONE.

[53] Bang K-S, Tak SH, Oh J, Yi J, Yu S-Y, Trung TQ (2017). Health Status and the Demand for Healthcare among the Elderly in the Rural Quoc-Oai District of Hanoi in Vietnam. BioMed Research International.

