# Supplementary material for: Reference data among general population and known-groups validity among hypertensive population of the EQ-5D-5L in Vietnam
Source: Qual Life Res. 2021 Aug 9;31(2):539–50. doi: 10.1007/s11136-021-02959-2 (PMC8847252; doi:10.1007/s11136-021-02959-2)
Supplement: Supplementary file 1 — Supplementary file1 (PDF 296 kb) [file 11136_2021_2959_MOESM1_ESM.pdf]

Title: Reference data among general population and known-groups validity among hypertensive population of the EQ-5D-5L in Vietnam
Journal name: Quality of Life Research

Author information:

1. Vu Quynh Mai, MSc (Corresponding author). [1]Center for Population Health Sciences, Hanoi University of Public Health; [2]Department of Epidemiology and Global Health, Umeå University.

   ORCID: 0000-0001-6962-0564

2. Assoc. Prof. Kim Bao Giang, MD, Ph.D., Hanoi Medical University.

   ORICD: 0000-0003-2290-0205

3. Prof. Hoang Van Minh, MD, Ph.D., Hanoi University of Public Health.

   ORICD: 0000-0002-4749-5536

4. Prof. Lars Lindholm, Ph.D., Department of Epidemiology and Global Health, Umeå University

   ORICD: 0000-0002-1633-2179

5. Sun Sun, Ph.D., [1]Department of Epidemiology and Global Health, Umeå University; [2] Research group Health Outcomes and Economic Evaluation, Department of Learning, Informatics, Management and Ethics, Karolinska Instiutet, Sweden

   ORICD: 0000-0001-5948-3025

6. Assoc. Prof. Klas Goran Sahlen, RN, MPH, Ph.D., Department of Epidemiology and Global Health, Umeå University

   ORICD: 0000-0002-3975-4868

Contact of the corresponding author:

Vu Quynh Mai, MSc. (last name underlined)

[1]Center for Population Health Sciences; Hanoi University of Public Health;

[2]Department of Epidemiology and Global Health, Umeå University

Email: vqm@huph.edu.vn/ mai.vu@umu.se Phone: (84) 971062521 ORCID: 0000-0001-6962-0564

Table 1a. Percentage of Females reporting the five dimensions by residence and age groups among general population.

	Urban						
	**18-24**	**25-34**	**35-44**	**45-54**	**55-64**	**65+**	**Total**
**Mobility**							
No problems	95	96.6	93.3	88.6	74.1	73.3	90
Slight problems	5	3.4	6.7	11.4	22.2	20	9.1
Moderate problems	0	0	0	0	0	6.7	0.5
Severe problems	0	0	0	0	3.7	0	0.5
**Self-care**							
No problems	100	100	97.8	100	96.3	93.3	98.6
Slight problems	0	0	0	0	3.7	6.7	0.9
Moderate problems	0	0	2.2	0	0	0	0.5
**Usual activities**							
No problems	92.5	100	97.8	97.1	96.3	80	95.9
Slight problems	7.5	0	2.2	2.9	3.7	20	4.1
**Pain/ Discomfort**							
No problems	65	70.7	60	62.9	44.4	46.7	61.4
Slight problems	35	25.9	33.3	31.4	44.4	33.3	32.7
Moderate problems	0	3.4	6.7	2.9	11.1	20	5.5
Severe problems	0	0	0	2.9	0	0	0.5
**Anxiety/ Depression**							
No problems	57.5	77.6	82.2	82.9	63	80	74.1
Slight problems	30	19	13.3	17.1	25.9	6.7	19.5
Moderate problems	12.5	1.7	2.2	0	11.1	13.3	5.5
Severe problems	0	0	2.2	0	0	0	0.5
Extreme problems	0	1.7	0	0	0	0	0.5
**Reporting full health**	40	50	48.9	51.4	37	33.3	45.5
	**Rural**						
**Mobility**							
No problems	100	96.6	93.4	75	74.4	71	88.5
Slight problems	0	3.4	5.3	23.5	23.1	25.8	10.5
Moderate problems	0	0	0	0	2.6	3.2	0.5
Severe problems	0	0	1.3	1.5	0	0	0.5
**Self-care**							
No problems	100	99.1	98.7	97.1	89.7	93.5	97.4
Slight problems	0	0.9	1.3	2.9	10.3	6.5	2.6
**Usual activities**							
No problems	100	97.4	92.1	97.1	87.2	87.1	94.9
Slight problems	0	2.6	3.9	2.9	12.8	12.9	4.3
Moderate problems	0	0	3.9	0	0	0	0.8
**Pain/ Discomfort**							
No problems	78.7	78.6	67.1	33.8	38.5	38.7	61.5
Slight problems	21.3	19.7	31.6	48.5	51.3	45.2	32.4
Moderate problems	0	1.7	0	11.8	7.7	16.1	4.6
Severe problems	0	0	1.3	5.9	2.6	0	1.5
**Anxiety/ Depression**							
No problems	80.3	83.8	81.6	76.5	69.2	77.4	79.6
Slight problems	18	14.5	15.8	16.2	20.5	16.1	16.3
Moderate problems	1.6	0	1.3	4.4	2.6	6.5	2
Severe problems	0	1.7	1.3	2.9	7.7	0	2
**Reporting full health**	63.9	67.5	55.3	27.9	33.3	35.5	51.8

Note: Data presented in percentage

Table 1b. Percentage of Males reporting the five dimensions by residence and age groups among general population.

	Urban						
	**18-24**	**25-34**	**35-44**	**45-54**	**55-64**	**65+**	**Total**
**Mobility**							
No problems	100	96.6	96.9	89.3	92.6	88.9	95.6
Slight problems	0	3.4	3.1	10.7	7.4	11.1	4.4
**Self-care**							
No problems	100	100	100	96.4	96.3	100	99
Slight problems	0	0	0	3.6	3.7	0	1
**Usual activities**							
No problems	100	91.4	96.9	92.9	100	100	96.1
Slight problems	0	8.6	3.1	7.1	0	0	3.9
**Pain/ Discomfort**							
No problems	74	77.6	59.4	67.9	66.7	66.7	70.6
Slight problems	20	17.2	40.6	28.6	29.6	33.3	25.5
Moderate problems	6	5.2	0	3.6	0	0	3.4
Severe problems	0	0	0	0	3.7	0	0.5
**Anxiety/ Depression**							
No problems	80	86.2	71.9	85.7	81.5	100	82.4
Slight problems	20	13.8	25	14.3	18.5	0	17.2
Extreme problems	0	0	3.1	0	0	0	0.5
**Reporting full health**	62	62.1	50	60.7	55.6	66.7	59.3
	**Rural**						
**Mobility**							
No problems	97.1	94.4	91.7	87.5	84.1	54.5	89.8
Slight problems	2.9	5.6	6	8.9	13.6	36.4	8.4
Moderate problems	0	0	1.2	1.8	2.3	0	0.8
Severe problems	0	0	1.2	1.8	0	9.1	1
**Self-care**							
No problems	100	99.1	98.8	98.2	97.7	95.5	98.7
Slight problems	0	0.9	0	1.8	2.3	0	0.8
Moderate problems	0	0	1.2	0	0	4.5	0.5
**Usual activities**							
No problems	100	95.4	96.4	92.9	88.6	90.9	95
Slight problems	0	4.6	2.4	7.1	11.4	4.5	4.4
Moderate problems	0	0	1.2	0	0	0	0.3
Severe problems	0	0	0	0	0	4.5	0.3
**Pain/ Discomfort**							
No problems	82.6	81.5	69	50	61.4	45.5	70
Slight problems	14.5	18.5	26.2	46.4	31.8	36.4	26.1
Moderate problems	2.9	0	2.4	3.6	2.3	18.2	2.9
Severe problems	0	0	2.4	0	4.5	0	1
**Anxiety/ Depression**							
No problems	81.2	85.2	81	89.3	86.4	77.3	83.8
Slight problems	17.4	13.9	13.1	7.1	13.6	22.7	13.8
Moderate problems	1.4	0.9	4.8	1.8	0	0	1.8
Severe problems	0	0	1.2	1.8	0	0	0.5
**Reporting full health**	69.6	69.4	56	44.6	59.1	31.8	59.5

Note: Data presented in percentage

Table 1c. Percentage of hypertension groups reporting the five dimensions.

	Non-hypertensive	Diagnosed for hypertension	Un-diagnosed for hypertension	**Total**
**Mobility**				
No problems	92.5	83.2	92.1	89
Slight problems	5.5	10.9	6.6	7.7
Moderate problems	1.4	3.6	0	1.9
Severe problems	0.5	1.5	0.4	0.8
Unable to walk	0	0.8	0.8	0.5
**Self-care**				
No problems	99.7	95	98.8	97.8
Slight problems	0.3	3.6	0.8	1.6
Moderate problems	0	0.4	0.4	0.2
Severe problems	0	0.8	0	0.3
Unable to wash or dress	0	0.2	0	0.1
**Usual activities**				
No problems	99.3	95.2	97.9	97.5
Slight problems	0.7	3.1	1.2	1.7
Moderate problems	0	1	0.4	0.5
Severe problems	0	0.6	0.4	0.3
**Pain/ Discomfort**				
No problems	79.4	73.2	83.1	77.8
Slight problems	18.4	21	15.3	18.8
Moderate problems	1.9	3.6	1.2	2.4
Severe problems	0.3	2.3	0.4	1.1
**Anxiety/ Depression**				
No problems	90.3	83.6	88.4	87.5
Slight problems	8.8	13.8	9.5	10.8
Moderate problems	0.7	2.1	1.7	1.4
Severe problems	0.2	0.4	0.4	0.3
**Reporting full health**	71.9	62.7	71.9	68.5

Note: Data presented in percentage

Table 2a. EQ-VAS and EQ-5D-5L indexes of Females by residence group.

	Urban					Rural				
	n	EQ-VAS		EQ-5D-5L index		n	EQ-VAS		EQ-5D-5L index	
		Mean	SD	Mean	SD		Mean	SD	Mean	SD
Total	220	80.91	13.46	0.93	0.09	392	80.09	13.84	0.93	0.09
Age group**										
18-24	40 (18.2%)	83.50	10.45	0.93	0.07	61 (15.6%)	83.56	9.72	0.97	0.05
25-34	58 (26.4%)	83.66	9.95	0.95	0.06	117 (29.8%)	84.49	11.76	0.97	0.06
35-44	45 (20.5%)	81.87	13.80	0.94	0.08	76 (19.4%)	80.18	14.06	0.95	0.10
45-54	35 (15.9%)	82.17	12.29	0.94	0.08	68 (17.3%)	77.00	14.34	0.88	0.11
55-64	27 (12.3%)	70.74	18.33	0.89	0.12	39 (9.9%)	72.51	16.21	0.88	0.12
65+	15 (6.8%)	75.87	15.96	0.89	0.13	31 (7.9%)	72.74	15.27	0.89	0.12
Total		80.91	13.46	0.93	0.09		80.09	13.84	0.93	0.10
p-value		**0.01**		0.13			**0.00**		**0.00**	
Highest education**										
Primary and lower	24 (10.9%)	79.79	14.48	0.94	0.08	104 (26.5%)	77.34	16.88	0.91	0.12
Secondary	56 (25.5%)	79.57	18.03	0.93	0.11	135 (34.4%)	79.24	13.10	0.93	0.09
High school	83 (37.7%)	81.06	12.12	0.92	0.08	62 (15.8%)	82.50	10.03	0.96	0.07
Undergraduate and higher	57 (25.9%)	82.47	9.14	0.95	0.07	91 (23.2%)	82.86	12.66	0.95	0.07
Total		80.91	13.46	0.93	0.09		80.09	13.84	0.93	0.10
p-value		0.91		0.26			**0.04**		**0.00**	
Geographic regions**										
Northern mountains	22 (10.0%)	78.00	16.18	0.92	0.10	52 (13.3%)	79.81	11.80	0.92	0.09
Red River delta	47 (21.4%)	81.15	11.05	0.94	0.08	94 (24.0%)	82.10	10.69	0.95	0.07
Highlands	14 (6.4%)	75.00	11.60	0.89	0.14	27 (6.9%)	80.93	13.52	0.94	0.07
Central Coast	37 (16.8%)	82.65	12.06	0.94	0.07	94 (24.0%)	74.31	16.95	0.91	0.13
South-East	70 (31.8%)	81.81	11.53	0.93	0.08	41 (10.5%)	81.07	13.11	0.96	0.06
Mekong river delta	30 (13.6%)	81.17	19.99	0.95	0.09	84 (21.4%)	83.74	13.02	0.94	0.10
Total		80.91	13.46	0.93	0.09		80.09	13.84	0.93	0.10
p-value		0.33		0.58			**0.00**		0.17	

Ethnicity *										
Kinh (as majority)	210 (95.5%)	80.88	13.62	0.93	0.09	345 (88.0%)	80.62	13.87	0.94	0.09
Others	10 (4.5%)	81.50	10.01	0.93	0.08	47 (12.0%)	76.17	13.07	0.90	0.11
Total		80.91	13.46	0.93	0.09		80.09	13.84	0.93	0.10
p-value		0.98		0.54			**0.02**		**0.00**	
Religion*										
Having no religion	157 (71.4%)	80.54	14.33	0.93	0.09	323 (82.4%)	81.00	13.22	0.93	0.09
Having religion	63 (28.6%)	81.84	11.05	0.94	0.07	69 (17.6%)	75.84	15.86	0.93	0.11
Total		80.91	13.46	0.93	0.09		80.09	13.84	0.93	0.10
p-value		0.82		0.29			**0.01**		0.86	
Marital status**										
Single	41 (18.6%)	82.44	12.15	0.92	0.08	38 (9.7%)	81.97	12.39	0.97	0.04
Married	156 (70.9%)	80.47	13.97	0.93	0.09	319 (81.4%)	80.28	13.78	0.93	0.10
Separated/Widowed/Divorced	23 (10.5%)	81.13	12.44	0.94	0.10	35 (8.9%)	76.29	15.50	0.89	0.11
Total		80.91	13.46	0.93	0.09		80.09	13.84	0.93	0.10
p-value		0.74		0.57			0.25		**0.01**	
Occupation**										
Having paid work	132 (60.0%)	81.58	12.31	0.94	0.09	271 (69.1%)	80.86	13.69	0.94	0.09
Student/Retired/Housewives	87 (39.5%)	79.97	15.13	0.93	0.08	117 (29.8%)	78.66	13.78	0.92	0.10
Unemployed	1 (0.5%)	75.00		0.92		4 (1.0%)	70.00	21.60	0.87	0.21
Total		80.91	13.46	0.93	0.09		80.09	13.84	0.93	0.10
p-value		0.61		0.30			0.14		0.31	
Having health insurance*										
No	40 (18.2%)	82.85	13.87	0.93	0.10	97 (24.7%)	81.01	13.11	0.95	0.07
Yes	180 (81.8%)	80.48	13.37	0.93	0.08	295 (75.3%)	79.79	14.08	0.93	0.10
Total		80.91	13.46	0.93	0.09		80.09	13.84	0.93	0.10
p-value		0.29		0.51			0.52		**0.03**	

Notes*: *Results from Mann-Whitney tests**. Results from* Kruskal Wallis H-Tests. *SD: Standard Deviation; n number of individuals.*

Table 2b. EQ-VAS and EQ-5D-5L indexes of Males by residence group.

	Urban					Rural				
	n	EQ-VAS		EQ-5D-5L index		n	EQ-VAS		EQ-5D-5L index	
		Mean	SD	Mean	SD		Mean	SD	Mean	SD
Age group**										
18-24	50 (24.5%)	84.24	8.71	0.96	0.06	69 (18.0%)	84.30	11.79	0.97	0.05
25-34	58 (28.4%)	84.60	12.55	0.96	0.06	108 (28.2%)	84.46	10.02	0.97	0.05
35-44	32 (15.7%)	82.09	12.76	0.94	0.08	84 (21.9%)	82.96	14.05	0.94	0.10
45-54	28 (13.7%)	82.68	12.58	0.95	0.08	56 (14.6%)	76.70	13.73	0.93	0.09
55-64	27 (13.2%)	78.52	14.21	0.95	0.08	44 (11.5%)	77.09	14.70	0.93	0.10
65+	9 (4.4%)	77.89	12.20	0.96	0.06	22 (5.7%)	70.68	16.78	0.87	0.15
Total		82.76	12.05	0.96	0.07		81.33	13.40	0.95	0.09
p-value		0.33		0.88			**0.00**		**0.00**	
Highest education**										
Primary and lower	18 (8.8%)	83.11	12.72	0.95	0.06	63 (16.4%)	75.86	16.12	0.91	0.14
Secondary	38 (18.6%)	80.63	13.28	0.94	0.09	141 (36.8%)	80.31	13.49	0.94	0.08
High school	76 (37.3%)	83.08	11.44	0.96	0.06	92 (24.0%)	83.51	12.25	0.97	0.07
Undergraduate and higher	72 (35.3%)	83.44	11.98	0.95	0.06	87 (22.7%)	84.66	10.71	0.96	0.06
Total		82.76	12.05	0.96	0.07		81.33	13.40	0.95	0.09
p-value		0.69		0.45			**0.00**		**0.03**	
Geographic regions**										
Northern mountains	17 (8.3%)	78.24	11.85	0.96	0.07	55 (14.4%)	81.80	13.18	0.93	0.11
Red River delta	43 (21.1%)	84.44	12.80	0.96	0.07	86 (22.5%)	81.29	12.82	0.95	0.07
Highlands	13 (6.4%)	85.77	8.13	0.96	0.09	26 (6.8%)	79.04	14.70	0.94	0.09
Central Coast	36 (17.6%)	78.92	12.85	0.95	0.07	92 (24.0%)	78.99	15.15	0.94	0.11
South-East	67 (32.8%)	83.66	11.94	0.95	0.07	36 (9.4%)	82.69	11.41	0.96	0.08
Mekong river delta	28 (13.7%)	84.29	10.78	0.96	0.05	88 (23.0%)	83.65	12.30	0.96	0.06
Total		82.76	12.05	0.96	0.07		81.33	13.40	0.95	0.09
p-value		0.10		0.90			0.41		0.92	

Ethnicity *										
Kinh (as majority)	201 (98.5%)	82.66	12.09	0.96	0.07	349 (91.1%)	81.24	13.47	0.95	0.08
Others	3 (1.5%)	89.00	8.54	0.95	0.09	34 (8.9%)	82.24	12.79	0.94	0.13
Total		82.76	12.05	0.96	0.07		81.33	13.40	0.95	0.09
p-value		0.38		0.93			0.83		0.82	
Religion*										
Having no religion	157 (77.0%)	82.61	12.51	0.95	0.07	322 (84.1%)	81.45	13.04	0.95	0.09
Having religion	47 (23.0%)	83.26	10.48	0.96	0.07	61 (15.9%)	80.72	15.25	0.94	0.09
Total		82.76	12.05	0.96	0.07		81.33	13.40	0.95	0.09
p-value		0.95		0.70			0.91		0.15	
Marital status**										
Single	72 (35.3%)	84.64	9.52	0.96	0.06	100 (26.1%)	83.62	11.70	0.97	0.06
Married	126 (61.8%)	81.57	13.28	0.95	0.08	272 (71.0%)	80.91	13.66	0.94	0.09
Separated/Widowed/Divorced	6 (2.9%)	85.00	10.49	0.95	0.06	11 (2.9%)	70.91	16.40	0.89	0.17
Total		82.76	12.05	0.96	0.07		81.33	13.40	0.95	0.09
p-value		0.47		0.78			**0.03**		0.07	
Occupation**										
Having paid work	149 (73.0%)	82.90	12.37	0.95	0.07	323 (84.3%)	82.31	12.62	0.95	0.08
Student/Retired/Housewives	52 (25.5%)	82.21	11.52	0.96	0.06	41 (10.7%)	80.56	15.48	0.95	0.08
Unemployed	3 (1.5%)	85.00	5.00	0.97	0.05	19 (5.0%)	66.32	13.00	0.87	0.17
Total		82.76	12.05	0.96	0.07		81.33	13.40	0.95	0.09
p-value		0.85		0.74			**0.00**		0.15	
Having health insurance*										
No	34 (16.7%)	78.27	15.27	0.96	0.06	119 (31.1%)	79.72	13.72	0.96	0.07
Yes	170 (83.3%)	83.65	11.14	0.95	0.07	264 (68.9%)	82.06	13.21	0.94	0.09
Total		82.76	12.05	0.96	0.07		81.33	13.40	0.95	0.09
p-value		0.09		0.77			0.09		0.32	

Notes*: *Results from Mann-Whitney tests**. Results from* Kruskal Wallis H-Tests. *SD: Standard Deviation; n: number of individuals.*

Table 2c. Post-hoc analysis on difference of the EQ-VAS indexes among general population sample

Age group	**18-24**	**25-34**	**35-44**	**45-54**	**55-64**
25-34	0.39 (1.00)				
35-44	-2.22 (0.00)	-2.61 (0.25)			
45-54	-5.24 (0.00)	-5.63 (0.00)	-3.91 (0.25)		
55-64	-9.15 (0.00)	-9.54 (0.00)	-6.93 (0.00)	-3.91 (0.11)	
65+	-10.60 (0.00)	-10.99 (0.00)	-8.38 (0.00)	-5.36 (0.003)	-1.45 (1.00)
Highest education	**Primary and lower**	**Secondary**	**High school**		
Secondary	2.17 (0.35)				
High school	4.88 (0.00)	2.72 (0.05)			
Undergraduate and higher	5.76 (0.00)	3.59 (0.00)	0.87 (1.00)		
Geographic regions	**Northern mountains**	**Red River delta**	**Highlands**	**Central Coast**	**South-East**
Red River delta	1.95 (1.00)				
Highlands	-0.04 (1.00)	-1.99 (1.00)			
Central Coast	-2.29 (1.00)	-4.25 (0.00)	-2.26 (1.00)		
South-East	2.33 (1.00)	0.38 (1.00)	2.37 (1.00)	4.63 (0.00)	
Mekong river delta	3.33 (0.26)	1.39 (1.00)	3.37 (0.74)	5.63 (0.00)	1.00 (1.00)
Marital status	**Single**	**Married**			
Married	-2.77 (0.01)				
Separated/Widowed/Divorced	-5.79 (0.00)	-3.02 (0.18)			
Occupation	**Having paid work**	**Student/ Retired/ Housewives**			
Student/Retired/Housewives	-1.92 (0.09)				
Unemployed	-12.59 (0.00)	-10.67 (0.00)			

Notes: Results presented as mean difference of EQ-VAS (p-value) by pair.

Table 2d. Post-hoc analysis on difference of the EQ-5D-5L indexes among general population sample

Age group	**18-24**	**25-34**	**35-44**	**45-54**	**55-64**
25-34	0.00 (1.00)				
35-44	-0.02 (0.35)	-0.02 (0.05)			
45-54	-0.04 (0.35)	-0.05 (0.00)	-0.02 (0.05)		
55-64	-0.04 (0.00)	-0.05 (0.00)	-0.03 (0.01)	-0.01 (1.00)	
65+	-0.07 (0.00)	-0.07 (0.00)	-0.05 (0.00)	-0.02 (0.49)	-0.02 (1.00)
Highest education	**Primary and lower**	**Secondary**	**High school**		
Secondary	0.02 (0.09)				
High school	0.04 (0.00)	0.02 (0.04)			
Undergraduate and higher	0.04 (0.00)	0.02 (0.04)	-0.00 (1.00)		
Geographic regions	**Northern mountains**	**Red River delta**	**Highlands**	**Central Coast**	**South-East**
Red River delta	0.02 (059)				
Highlands	0.01 (1.00)	-0.01 (0.19)			
Central Coast	-0.00 (1.00)	-0.02 (0.19)	-0.01 (1.00)		
South-East	0.02 (0.68)	0.00 (1.00)	0.01 (1.00)	0.02 (0.26)	
Mekong river delta	0.02 (0.42)	0.00 (1.00)	0.01 (1.00)	0.02 (0.13)	0.00 (1.00)
Marital status	**Single**	**Married**			
Married	-0.02 (0.01)				
Separated/Widowed/Divorced	-0.05 (0.00)	-0.03 (0.02)			
Occupation	**Having paid work**	**Student/ Retired/ Housewives**			
Student/Retired/Housewives	-0.01 (0.22)				
Unemployed	-0.06 (0.00)	-0.05 (0.02)			

Notes: Results presented as mean difference of EQ-5D-5L indexes (p-value) by pair.

Table 3a. Post-hoc analysis on difference of the EQ-VAS among CH2' sample

	Non-hypertensive group		Diagnosed for hypertension group	
**Age group**			**40-49**	**50-59**
50-59			-3.00 (0.34)	
60+			-6.00 (0.01)	-3.00 (0.39)
**BMI level**	**Underweight**	**Normal**		
18.5-24.9 (normal)	6.00 (0.02)			
>25 (overweight/ obese)	8.00 (0.00)	2.00 (0.59)		

Notes: Results presented as mean difference of EQ-VAS (p-value) by pair.

Table 3b. Post-hoc analysis on difference of the EQ-5D-5L indexes among CH2' sample

	Non-hypertensive group		Diagnosed for hypertension group		Undiagnosed for hypertension group	
**Age group**	**40-49**	**50-59**				
50-59	-0.02 (0.03)					
60+	-0.01 (1.00)	0.01 (0.74)				
**Highest education**	**Primary**	**Secondary**	**Primary**	**Secondary**		
Secondary	0.01 (0.13)		0.03 (0.09)			
High school or higher	0.02 (0.04)	0.00 (1.00)	0.03 (0.15)	-0.00 (1.00)		
**Marital status**					**Single**	**Married**
Married					0.01 (1.00)	
Separate/divorce/widow					-0.06 (1.00)	-0.07 (0.00)

Notes: Results presented as mean difference of EQ-5D-5L indexes (p-value) by pair.
